# A Randomized, Double-Blind, Placebo-Controlled Decentralized Trial to Assess Sleep, Health Outcomes, and Overall Well-Being in Healthy Adults Reporting Disturbed Sleep, Taking a Melatonin-Free Supplement

**DOI:** 10.3390/nu15173788

**Published:** 2023-08-30

**Authors:** Antonija Kolobaric, Susan J. Hewlings, Corey Bryant, Christopher S. Colwell, Christopher R. D’Adamo, Bernard Rosner, Jeff Chen, Emily K. Pauli

**Affiliations:** 1 Radicle Science, Inc., Del Mar, CA 92014, USA; antonija.kolobaric@radiclescience.com (A.K.); corey.bryant@radiclescience.com (C.B.); jeff@radiclescience.com (J.C.); emily@radiclescience.com (E.K.P.); 2 Center for Neuroscience, University of Pittsburgh, Pittsburgh, PA 15260, USA; 3 Department of Psychiatry & Biobehavioral Sciences, University of California–Los Angeles, Los Angeles, CA 90095, USA; ccolwell@mednet.ucla.edu; 4 Department of Family and Community Medicine, University of Maryland School of Medicine, Baltimore, MD 21201, USA; cdadamo@som.umaryland.edu; 5 Department of Biostatistics, Harvard T.H. Chan School of Public Health, Boston, MA 02115, USA; stbar@channing.harvard.edu

**Keywords:** sleep, botanical synergy, cannabinoids, health related quality of life, PROMIS

## Abstract

Inadequate sleep is a global health concern. Sleep is multidimensional and complex; new multi-ingredient agents are needed. This study assessed the comparative effects of two multi-ingredient supplements on sleep relative to placebo. Adults (N = 620) seeking better sleep were randomly assigned to receive one of three study products. Sleep A (contained lower (0.35 mg THC and higher levels of botanicals (75 mg each hops oil and valerian oil), Sleep B (contained higher THC (0.85 mg) and lower botanicals (20 mg each hops oil and valerian oil) or placebo) for 4 weeks. Sleep disturbance was assessed at baseline and weekly using NIH’s Patient-Reported Outcomes Measurement Information System (PROMIS™) Sleep Disturbance SF 8A survey. Anxiety, stress, pain, and well-being were assessed using validated measures at baseline and weekly. A linear mixed-effects regression model was used to assess the change in health outcome score between active product groups and the placebo. There was a significant difference in sleep disturbance, anxiety, stress, and well-being between Sleep A and placebo. There was no significant difference in any health parameter between Sleep B and placebo. Side effects were mild or moderate. There were no significant differences in the frequency of side effects between the study groups. A botanical blend containing a low concentration of THC improved sleep disturbance, anxiety, stress, and well-being in healthy individuals that reported better sleep as a primary health concern.

## 1. Introduction

Inadequate sleep became a global public health concern, leading to greater awareness of the negative impact from a lack of sleep. Inadequate sleep is related to increased obesity and inflammation, impairs immune and antioxidant defenses, and negatively impacts mood [1,2]. Inadequate sleep is associated with heightened emotional reactivity, reduced attention, and impaired memory and cognitive function [3]. Individuals who are sleep deprived are less productive and report a lower overall quality of life [4]. It is suggested that shorter sleep duration alters the gut microbiome. In turn, these changes in gut microbiome may drive increases in systemic inflammation associated with metabolic syndrome and many other lifestyle-related conditions [5].

It is recommended that anyone suffering with sleep disturbances consult a health professional and consider a multidimensional assessment, recognizing that an intervention that targets sleep onset may not accurately address sleep latency. Furthermore, because sleep involves multiple mechanisms, it may be advantageous to consider a multi-ingredient therapeutic approach targeting a variety of pathways to support sleep. There are many treatments aimed at improving sleep, including behavioral management, lifestyle management, exercise, diet, pharmacological interventions, and dietary supplements [6]. Many individuals experience negative side effects from prescribed medications and choose to seek alternate solutions, such as dietary supplements and plant-based alternatives [7].

Cannabinoids are a potential plant-based alternative to prescription products for improving sleep. The cannabis plant is composed of 120 different phytocannabinoids. Delta-9 tetrahydrocannabinol (THC) and cannabidiol (CBD) are perhaps the most widely known and researched, but others, such as cannabinol (CBN) are growing in popularity [8,9]. Cannabinoids produce varying effects in the human body by interacting with the endocannabinoid system (ECS), which is located throughout the brain and the central and peripheral nervous system [8]. The ECS was suggested to modulate the circadian sleep/wake cycle. Therefore, the role of cannabinoids in sleep modulation is supported by the role of the endocannabinoid system in circadian regulation [9]. A recent systematic review, including 14 preclinical and 12 clinical studies, concluded “there are promising signs in a number of therapeutic applications that warrant additional study and there is a clear need for intensification of high-quality research into the safety and efficacy of cannabinoid therapies for treating sleep disorders…” [10]. CBD is non-intoxicating and was shown to be safe and well-tolerated in humans, even at very high doses (e.g., 1500 mg twice daily for six days or as an acute dose of 6000 mg [11]). CBN is a by-product of THC and is found in small amounts in the cannabis plant. CBN gained consumer interest as an ingredient to benefit sleep [12,13]. However, research related to CBN and sleep is limited, and the majority of it dates back to the 1970s and 1980s [14].

Other non-pharmacological alternatives were investigated. For example, γ-aminobutyric acid (GABA) is a non-protein amino acid, well known for its role as an inhibitory neurotransmitter in the central nervous system, promoting relaxation and sleep [15,16]. Additionally, γ-Glutamylethylamine, also known as L-theanine, is a non-protein amino acid found in green tea, often used to improve sleep and modify stress. It is thought to increase expression of dopamine and serotonin in the brain by increasing GABA levels [17,18]. It was suggested that these compounds provide synergistic benefits. In mice, GABA/L-theanine mixture (100/20 mg/kg) demonstrated a decrease in sleep latency (20.7 and 14.9%) and an increase in sleep duration (87.3 and 26.8%) compared to GABA or L-theanine alone [18].

Botanical essential oils are also gaining popularity for their effectiveness in improving sleep. Hops are herbaceous perennial plants belonging to the class Magnoliopsida, subclass Hamamelididae, order Urticales, family Cannabaceae, genus Humulus. It was known for centuries for its many health benefits, including its sedative effects [19]. Several studies support the sedative properties of hops oil [20,21]. The sedative effect is thought to occur due to a degradation product, 2-methyl-3-buten-2-ol, that increases GABA [22]. Alone, the amount of any one botanical is most likely too low to lead to any significant benefit, but it is believed that the synergy with other phytocompounds produces the effect [23,24].

Another popular oil often used together with hops, is *Valeriana* L., a group of perennial herbs belonging to the family Caprifoliaceae. It was used for centuries around the globe to improve sleep. The roots and rhizomes are most often used for medicinal benefits. There are more than 200 species of *Valeriana*, with *Valeriana officinalis* L. used most often in US products. In mice, the essential oil was determined to be the active part of the plant. The compounds within the oils could act synergistically on GABA receptors to increase GABA release and inhibit uptake [25,26,27].

Though each of the botanicals discussed thus far showed promise for improving sleep, it is intriguing to consider the concept of synergy, especially because sleep is multidimensional and complex, thus warranting a multi-ingredient approach. The discussion of botanical synergy is not new. In recent years, it is commonly called “the entourage effect” when discussed in reference to the potential therapeutic effects of the synergy between phytocannabinoids and the many other compounds found in the cannabis plant [28]. Demonstrated by several review papers, the application of botanical synergy extends beyond the cannabis plant to other botanicals [29,30]. It was suggested that synergy among the cannabinoid compounds may enhance the effectiveness of THC, thus allowing a lower effective dose, minimizing the psychoactive effects of THC while maintaining the benefits [31]. Whether compounds from other botanicals would provide a similar synergistic effect is unknown.

The aim of this study was to assess the effects of two different softgel dietary supplements, one with lower THC and higher levels of other botanicals (Sleep A), and one with higher THC and lower levels of other botanicals (Sleep B), on sleep disturbance relative to placebo.

## 2. Materials and Methods

This study, Radicle^TM^ Rest, was a randomized, double-blind, placebo-controlled, parallel-group virtual trial, designed to assess the effects of health and wellness products on sleep, anxiety, stress, pain, and overall health-related quality of life. Participants were not required to attend in-person visits. All data were collected via online surveys, which participants accessed via participant-specific hyperlinks sent to them at scheduled times through their preferred means of communication (email or SMS text). Participants were recruited online from across the US through social media, Radicle Science’s electronic mailing list, and a third-party consumer network with nationwide representation. Recruitment emails containing links to the study screener were sent to those within the Radicle Science mailing list and consumer network, while social media advertisements led to a study landing page with a link to the study screener. Participants were eligible if they were 21 years old or older, resided in the United States, expressed a desire for better sleep, and ranked their desire for better sleep as a primary reason for taking a dietary supplement. Individuals were excluded if they were pregnant or breastfeeding, or taking medications that posed a health risk when used in conjunction with any of the study product ingredients. Subjects reported that they were healthy but were not screened for any diagnosed conditions. To best represent the real world, a diagnosed sleep disorder was not a factor in eligibility; instead, the information was ascertained during intake. Eligible individuals were directed to a secure online portal to provide informed consent. Participants indicated their consent electronically by signing the informed consent form and were sent a digital copy of the electronic consent. Eligible individuals were advised to consult with their healthcare provider before participating if they had a diagnosed medical condition, were on any prescription medication or supplements, or had any upcoming medical procedures planned. Immediately following informed consent, participants completed an intake survey, which collected basic demographic information, health behaviors, and experienced sleep quality.

**Figure 1:**
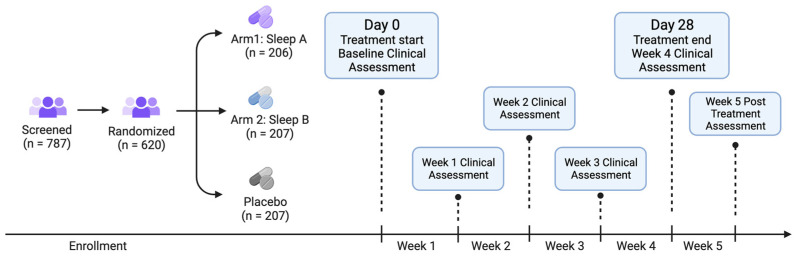
Study flow diagram. Eligible participants were enrolled in the study and randomized into one of three groups: Sleep A, Sleep B, or placebo. We collected baseline clinical measures before participants started using their study product. Participants used study product for 4 weeks total. Clinical and other measures were collected at the end each week as well as 1 week post study product use.

Recruits who consented to participate and completed intake were randomized to one of three study arms (see below for details on randomization): Sleep A, Sleep B, or placebo (Figure 1).

Participants were sent a 4-week supply of their study product in the mail, along with the product insert, detailing instructions for study participation. All study products were provided by the partnering manufacturer and analyzed at an independent laboratory to ensure active ingredient identification, safety, and potency. Participants were instructed to take one softgel 30 min before bedtime, and informed that they could escalate to a maximum of four softgels per day as needed throughout the study duration. The number of softgels consumed were recorded. Participants were directed to wait 5 days before increasing the number of softgels taken. The study product formulations are proprietary to the manufacturer, but both Sleep A and B formulations contained the same amount of CBD, CBN, and L-theanine; Sleep A formula contained lower amounts of THC (0.35 mg) and higher amounts of GABA (150 mg), hops oil (75 mg), and valerian oil (75 mg) relative to the Sleep B formula (0.85 mg THC, 125 mg GABA, 20 mg hops oil, 20 mg valerian oil). The study was double-blind; neither the participants nor those who collected and analyzed the data were aware of the product participants received until the conclusion of the study. The study was conducted from October 2022 to December 2022.

For 5 weeks following the study initiation and baseline (4 weeks taking the study product and 1 week after finishing the study product), participants were asked to complete online surveys, which they accessed via unique hyperlinks sent at scheduled times via email or text. During the baseline week, participants completed health outcome assessments of their sleep, feelings of anxiety, stress, pain, and overall well-being, using validated, patient-reported outcome measures (Table 1).

Throughout the study duration, participants received a health survey asking them to report their study product usage for the week and health outcome assessments for their sleep disturbance, feelings of anxiety, stress, pain, and overall well-being from the past week using the same validated health measures used at baseline. In every study survey, following receipt of their product, participants were also prompted to report any side effects and were encouraged to contact the research team directly if they experienced side effects at any point.

The Sterling Independent Review Board (SIRB) approved the study [10147]. The master protocol Radicle Rest^TM^ was registered on ClinicalTrials.gov [Identifier: NCT05511818]. It should be noted that the protocol registered is not this specific study, but rather is the templated study protocol utilized for all Radicle Rest studies.

### 2.1. Randomization

Participants were randomly assigned to one of the three study product arms, with an equal chance of being assigned to each group (1:1:1 ratio). Prior to randomization, participants were stratified by their assigned sex at birth (male, female) then randomized to one of the study arms using the randomizer with evenly presented elements in the Qualtrics^®^ XM platform (Provo, UT, USA).

### 2.2. Outcomes

Radicle^TM^ Rest is a templated trial protocol incorporating validated assessments that received overall IRB approval that was then applied to each individual study. The study design and assessments used do not change from study to study, only the actives and placebos change. The primary outcomes were changed in the PROMIS™ Sleep Disturbance 8A scale [32] as well as the odds of achieving a minimal clinically important difference (MCID). MCID was defined as a reduction that is greater than or equal to one-half the standard deviation of the baseline score [33]. The MCID standard deviation criterion was calculated by study arm. Secondary outcomes included changes in anxiety, stress, pain, and overall wellbeing. Secondary outcomes were assessed using PROMIS™ Anxiety 4a, PROMIS™ Stress 4a, PEG (pain, enjoyment, general activity), and the World Health Organization (WHO)-5 Well-being index.

### 2.3. Safety

The frequency of spontaneously reported side effects and their severity were assessed. Severity was determined based on reported utilization of medical services in response to the side effects according to the following grading schema based on the Common Terminology Criteria for Adverse Events (CTCAE; v5.0 USDHHS): mild: no intervention (medication or medical advice) needed; moderate: a medication was taken due to the side effect or a participant sought medical advice from their HCP, urgent clinic or ED; severe: the side effect was medically significant but not life-threatening and/or the participant was admitted to the hospital for care and attention; life threatening: immediate medical intervention required and the participant was hospitalized, placed in the intensive care unit due the side effect, and/or suffered long-lasting negative effects as a result of the side effect.

### 2.4. Covariates

Prior to analysis, we collapsed three demographic variables, including race, education, and ethnicity. Race was recoded as white (including participants who identified as white), non-white (including participants who identified as Black, multi-racial, Asian, unknown, prefer not to say, some other race, or American Indian or Alaska Native), and Native Hawaiian or Pacific Islander (including participants who identified as Native Hawaiian or Pacific Islander). Education was recoded as college degree (including participants who have a bachelor’s or associate degree, and masters or professional degree), and no college degree (including participants who selected prefer not to say, less than high school, trade/technical/vocational degree, high school diploma no college, and some college no degree). Ethnicity was recoded to Hispanic (including participants who selected yes) and Non-Hispanic (including participants who selected no, or prefer not to say). We adjusted for baseline demographics, including age, recoded race, recoded ethnicity, recoded education level, sex assigned at birth (male, female), and body mass index (BMI; calculated through self-reported height and weight).

### 2.5. Power Analysis

A power analysis was conducted to ensure sufficient power to detect a significant difference in the change in the PROMIS™ Sleep Disturbance 8A scale for each study product arm relative to placebo. A sample size of 190 for each study group would yield 90% power to find a difference in mean change between each study product arm versus the placebo arm at a two-sided *p* value of 0.05 corrected for multiple comparisons (Bonferroni). Recruiting up to 300 participants per study arm would allow us to maintain adequate sample size under anticipated attrition levels (45%).

### 2.6. Statistical Analysis

A linear, mixed-effects regression model was used to assess the differences in the change in the variables of interest between each active product arm versus placebo. The parameter “na.action = na.omit” was set for each model, meaning that participants were excluded only from those models for which they did not have available data. All models were fit using an unstructured covariance matrix with a random intercept at the individual level, and a random slope and intercept at the study week level. The models tested the difference in the interaction between product arm and study week for active arm versus placebo, controlling for sex, age, race, ethnicity, and BMI. Post hoc Bonferroni-adjusted pairwise comparisons were used to assess the differences in the odds of achieving a MCID for sleep between each active product arm placebo, controlling for sex, age, race, ethnicity, education, and BMI.

### 2.7. Software

The Python programming language, version 3.95 (packages: pandas, version 1.4.3, and numpy, version 1.20.2) were used for data processing. R, version 4.2.3 (packages: nlme, version 3.1-162, marginal effects, version 0.11.1, and tidyverse, version 2.0.0) was used to conduct the statistical analyses, and package table one version 0.13.2 was used to create table one.

## 3. Results

### 3.1. Particpants

The study population reflects well the population of US consumers that elect to purchase health and wellness supplements. At baseline, 90% of participants reported that they suffered from stress and 76% reported that they suffered from sleep disturbances most (63.6% reported mild or moderate sleep disturbances). In this study, 66% of participants were female, 34% were male, and 80% identified their race as white. After stratification, 206 participants were randomly assigned to Sleep A, 207 to Sleep B, and 207 to placebo. The groups did not significantly differ on any demographic or outcomes variables at baseline (Table 2). The amount of softgels taken by each study arm were recorded and analyzed and not significantly different between the groups, as shown by a single factor one way ANOVA: F (2, 256) = 1.527, *p* = 0.219. Further, trends in product consumption were roughly equivalent across arms, with average daily dosages slightly increasing throughout the study.

### 3.2. Sleep Quality

**Figure 2:**
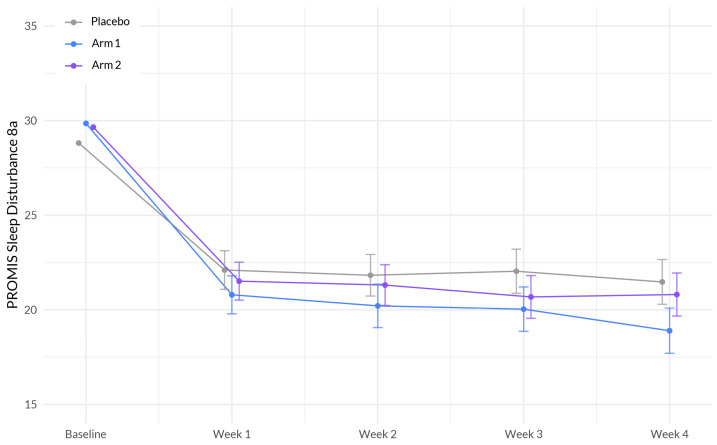
Evolution of PROMIS sleep disturbance 8a between the three arms during the study period. The plot illustrates the interaction between treatment (Sleep A; Arm 1 and Sleep B; Arm 2) and week on the sleep disturbance scale based on a linear mixed-effects model. The *x*-axis represents the weeks of the study, while the *y*-axis represents the outcome scale. The lines represent the trajectories of sleep disturbance for each treatment arm over time. The plot highlights the nature of treatment effects on sleep quality, as captured by the linear mixed-effects model, allowing for the incorporation of random effects and accounting for within-subject correlations.

The interaction between study week and Sleep A (Arm 1) showed a significant negative association with sleep disturbance (β = −0.639, *p* = 0.0027). This indicates that the effect of study week on sleep disturbance differed between the treatment groups, with participants in Sleep A (Arm 1) experiencing a greater reduction in sleep disturbance over time compared to the placebo group. Education demonstrated a significant positive association with sleep disturbance (β = 1.846, *p* < 0.0001), suggesting that individuals without a college degree reported higher levels of sleep disturbance. BMI exhibited a significant positive association with sleep disturbance (β = 0.070, *p* = 0.0163), indicating that higher BMI was associated with higher levels of sleep disturbance (Figure 2, Table 3). We did not observe any significant differences in the likelihood of achieving a minimum clinically important difference (MCID) in between Sleep A (estimate = 1.33, 95% CI [0.85, 1.81], *p* = 0.242) or Sleep B (estimate = 1.23, 95% CI [0.79, 1.67], *p* = 0.477) and placebo (42.2%). MCID is defined as a change in one half the standard deviation of the baseline score.

### 3.3. Anxiety

**Figure 3:**
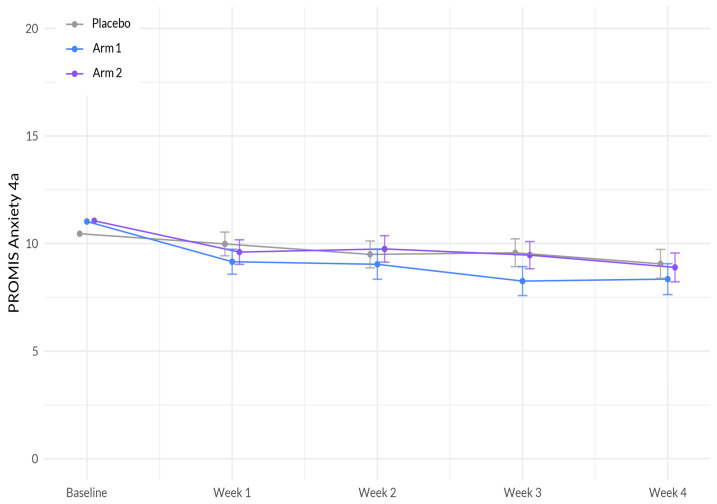
Evolution of PROMIS Anxiety 4a scores between the three arms during the study period. The plot illustrates the interaction between treatment (Sleep A; Arm 1 and Sleep B; Arm 2) and week on the anxiety scale, based on a linear mixed-effects model. The *x*-axis represents the weeks of the study, while the *y*-axis represents the outcome scale. The lines represent the trajectories of anxiety for each treatment arm over time. The plot highlights the nature of treatment effects on anxiety as captured by the linear mixed-effects model, allowing for the incorporation of random effects and accounting for within-subject correlations.

The analysis revealed several significant associations with anxiety. First, there was a significant negative interaction between study week and Sleep A (Arm 1) (β = −0.258, *p* = 0.041), indicating that as the study progressed, participants using Sleep A were more likely to experience a decrease in anxiety compared to participants using placebo (Figure 3, Table 4). Additionally, education showed a significant positive association with anxiety (β = 0.744, *p* = 0.005), suggesting that individuals with a higher level of education experienced higher levels of anxiety. Lastly, age demonstrated a significant negative association with anxiety (β = −0.035, *p* = 0.005), indicating that as age increased, anxiety levels tended to decrease.

### 3.4. Stress

**Figure 4:**
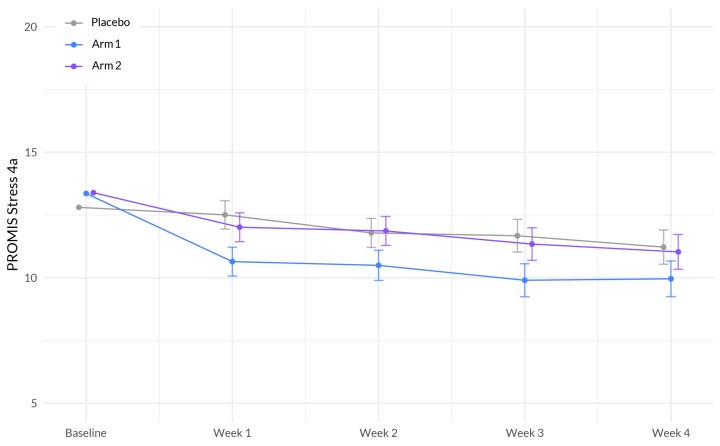
Evolution of PROMIS Stress 4a between the three-arms during the study period. The plot illustrates the interaction between treatment (Sleep A; Arm 1 and Sleep B; Arm 2) and week on the stress scale, based on a linear mixed-effects model. The *x*-axis represents the weeks of the study, while the *y*-axis represents the outcome scale. The lines represent the trajectories of stress for each treatment arm over time. The plot highlights the nature of treatment effects on stress as captured by the linear mixed-effects model, allowing for the incorporation of random effects and accounting for within-subject correlations.

The interaction between study week and Sleep A (Arm 1) showed a significant negative association with stress (β = −0.360, *p* = 0.004). This indicates that the effect of study week on stress levels differed between the treatment groups, with participants in Sleep A (Arm 1) showing a larger decrease in stress over time compared to the placebo group (Figure 4, Table 5). Additionally, sex demonstrated a significant negative association with stress (β = −0.666, *p* = 0.019), suggesting that males reported higher levels of stress compared to females. Age showed a significant negative association with stress (β = −0.065, *p* < 0.001), indicating that older participants reported lower levels of stress. Furthermore, BMI exhibited a significant positive association with stress (β = 0.033, *p* = 0.049), indicating that higher BMI was associated with higher stress levels.

### 3.5. Pain

**Figure 5:**
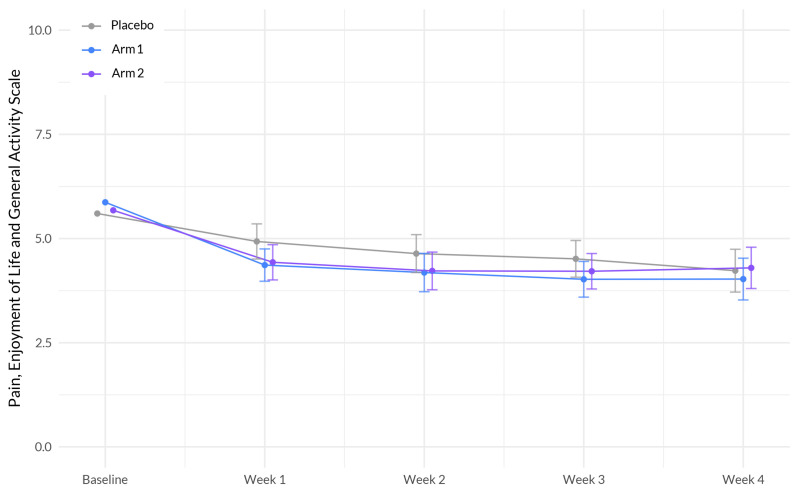
Evolution of PEG scores between the three arms during the study period x. The plot illustrates the interaction between treatment (Sleep A; Arm 1 and Sleep B; Arm 2) and week on EG scores, based on a linear mixed-effects model. The *x*-axis represents the weeks of the study, while the *y*-axis represents the outcome scale. The lines represent the trajectories of PEG scores for each treatment arm over time. The plot highlights the nature of treatment effects on PEG scores as captured by the linear mixed-effects model, allowing for the incorporation of random effects and accounting for within-subject correlations.

Our primary analyses revealed no significant differences in the rate of mean PEG (pain, enjoyment, and general activity) score change between Sleep A and placebo (β = −0.024, *p* = 0.788), or between Sleep B and placebo (β = 0.032, *p* = 0.713), see Figure 5 and Table 6. However, age showed a significant positive association with pain (β = 0.024, *p* = 0.019), indicating that as age increased, participants reported higher levels of pain. Education also demonstrated a significant positive association with pain (β = 0.706, *p* = 0.0023), suggesting that individuals with a higher level of education experienced higher levels of pain. Additionally, study week showed a significant negative association with pain (β = −0.307, *p* < 0.001), indicating that as the study progressed, participants reported lower levels of pain.

### 3.6. Overall Well-Being

**Figure 6:**
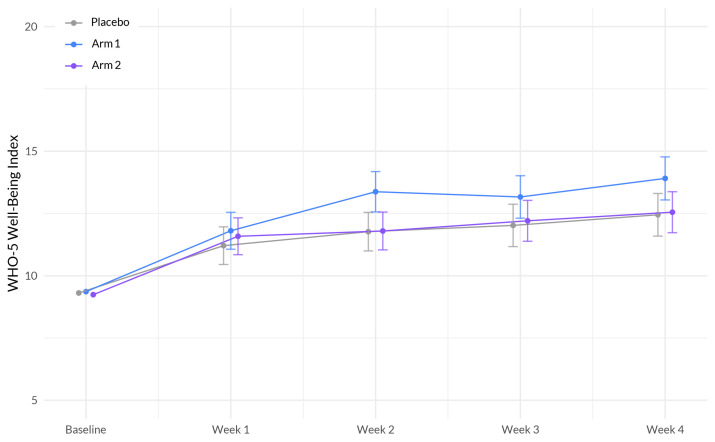
Evolution of WHO well-being scores between the three arms during the study period x. The plot illustrates the interaction between treatment (Sleep A; Arm 1 and Sleep B; Arm 2) and week on the well-being scale, based on a linear mixed-effects model. The *x*-axis represents the weeks of the study, while the *y*-axis represents the outcome scale. The lines represent the trajectories of well-being for each treatment arm over time. The plot highlights the nature of treatment effects on well-being as captured by the linear mixed-effects model, allowing for the incorporation of random effects and accounting for within-subject correlations.

The interaction between study week and Sleep A (Arm 1) showed a significant positive association with well-being (β = 0.318, *p* = 0.0346). This indicates that the effect of study week on overall well-being differed between the treatment groups, with participants in Sleep A (Arm 1) experiencing a greater improvement in well-being over time compared to the placebo group. Education demonstrated a significant negative association with well-being (β = −1.559, *p* < 0.001), suggesting that individuals without a college degree reported lower levels of overall well-being. BMI exhibited a significant negative association with well-being (β = −0.060, *p* = 0.0061), indicating that higher BMI was associated with lower levels of well-being (Figure 6, Table 7).

### 3.7. Side Effects

**Figure 7:**
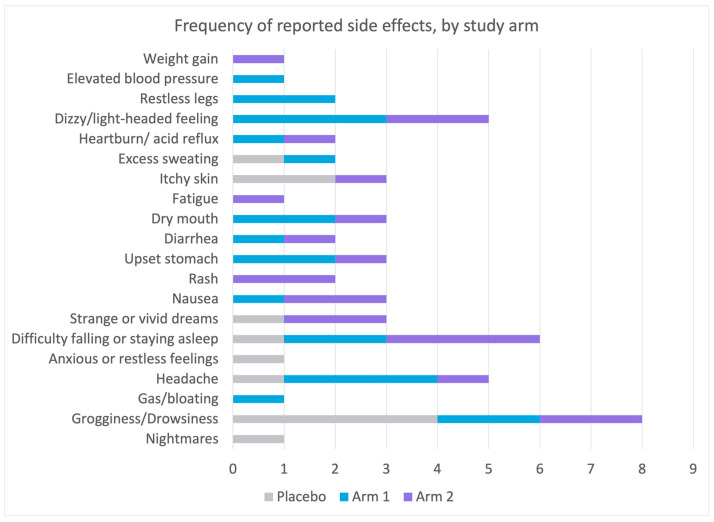
Comparison of side effects between active arms (Sleep A; Arm 1 and Sleep B; Arm 2) and placebo. This figure displays the occurrence of side effects in the active arms (Sleep A; Arm 1 and Sleep B; Arm 2) compared to the placebo group. Participants with sleep disturbance received Sleep A (Arm 1), Sleep B (Arm 2), or placebo for 4 weeks. Side effects, primarily grogginess and drowsiness, were slightly more common in the active arms but were mild and non-serious, requiring no emergency or non-emergency healthcare services.

Side effects were slightly more common among the active arms (Arm 1 and Arm 2, (χ^2^(2) = 5.64, *p* = 0.059)), predominantly grogginess and drowsiness and mostly mild; none were considered serious or required use of emergency or non-emergency healthcare services (Figure 7).

## 4. Discussion

We presented a randomized, double-blinded, placebo-controlled trial to evaluate the effectiveness and safety of two orally ingested softgel dietary supplements, Sleep A and Sleep B, compared to a placebo over four weeks of treatment. We observed a significant difference in effect on four health outcomes (sleep disturbance, anxiety, stress, and overall well-being) between Sleep A formula and placebo control. We observed no significant difference in effect on any health outcomes between Sleep B formula and placebo control. Importantly, both supplements exhibited favorable safety profiles, as all side effects were mild or moderate and there were no significant differences in the frequency of reported side effects between the active and placebo arms.

We hypothesize that the change in the quality of sleep between the baseline and the first week of treatment is directly related to the use of study products, meaning that the placebo effect was the reason for the increase in both groups. The change in placebo group remained consistent over the course of the study, while the treatment group continued to improve. This is consistent with the literature investigating the change in placebo effect over time, which suggests that the change remains constant over the course of a study lasting as long as 12 months, especially in study designs utilizing subjective outcome measures [34].

This study is among the first to investigate the effectiveness and safety of supplements that aim to enhance sleep and overall health in a large participant sample. These supplements were specifically designed to harness the potential synergistic effects of botanical ingredients that demonstrated favorable outcomes in promoting better sleep. Our primary analyses indicate that over the course of four weeks using the study product, the Sleep A group experienced a significantly greater reduction in sleep disturbance compared to the placebo group. Additionally, participants in the Sleep A group also showed significantly greater improvements in anxiety, stress, and overall well-being compared to those in the placebo group. These results are expected, given the intricate connections between sleep, anxiety, stress, and overall well-being [35].

Interestingly, we observed no significant differences in any of the health outcomes between Sleep B and placebo. It is important to note that both Sleep A and B formulas contained the same amount of CBD, CBN, and L-Theanine, while Sleep formula A contained a lower amount of THC and higher amounts of GABA, hops oil, and valerian oil than the Sleep B formula. Since the current study did not employ a factorial design to examine the individual effects of all ingredients, as well as their potential interactions, we are unable to determine the main driver(s) between the different impacts Sleep A and Sleep B had on behavioral outcomes. We put forward a hypothesis regarding the potential reasons for the superior performance of Sleep A in reducing sleep disturbances compared to Sleep B. There are two primary factors that we believe contribute to this outcome. Firstly, Sleep A contains a lower amount of THC compared to Sleep B. One study found that those participants who use cannabis multiple times a week for its sleeping effects prefer strains with lower amounts of THC, suggesting that the strains with lower THC may be more effective at promoting sleep [36]. Secondly, Sleep A contains higher levels of three specific ingredients: GABA, hops oil, and valerian oil. These ingredients are known to function as positive modulators of GABA, a neurotransmitter associated with promoting sleep and inducing mild sedation [23,37]. Therefore, it is plausible that the presence of these ingredients in Sleep A contributes to its effectiveness in improving sleep quality.

The data obtained in the present study are consistent with previous findings that a combination of cannabinoids could improve sleep. For example, a randomized, controlled crossover trial administering a combination product, containing THC 20 mg/mL, CBN 2 mg/mL, CBD 1 mg/mL, and naturally occurring terpenes (extracted from the cannabis plant) in pharmaceutical grade sunflower oil for 2 weeks, demonstrated an improvement in sleep quality in subjects with insomnia when compared to placebo [13]. Similarly, subjects receiving a tablet containing 10 mg THC and 5 mg CBN nightly experienced significantly improved sleep quality and slept significantly longer, with a 5% increase in sleep duration [12]. We recently published the results of a similar sleep study on 1793 adults [38]. Participants were randomly assigned to take 1 of 6 products, containing either 15 mg CBD or 5 mg melatonin, alone or in combination with minor cannabinoids, including CBN. Most participants (56% to 75%) across all formulations experienced a clinically important improvement in their sleep quality, though not statistically better than the active control group that took 5 mg of melatonin alone.

This study was intended to approximate the “real world” effectiveness of the study products by administering them to a broad population that used the products in a manner and setting similar to that of actual consumers. Unlike conventional clinical trials, which often have restrictive eligibility criteria and rigorous monitoring, the data in question may exhibit higher levels of missingness and heterogeneity. Nonetheless, conventional clinical trials involving natural products frequently suffer from limited sample sizes and lack external validity, as the participants’ characteristics and behaviors may not accurately represent those of real-world users. Consequently, studies such as this try to reflect the real-world effects of such products and possess distinct value in their capacity to provide evidence for regulatory and clinical decision-making and additional clinical trial design [39].

Whether or not the results of our study reflect botanical synergy, meaning that the higher levels of the hops, valerian, and GABA allowed for the lower level of THC to be effective, was not directly investigated in this study. However, it poses interesting questions and warrants further investigation.

This study has multiple limitations. First, approximately 26% of participants did not complete any follow-up surveys. However, the overall attrition level was still below our anticipated attrition (45%) and the study remained adequately powered to detect significant sleep changes. Furthermore, because the products used in this study were combinatorial, we are unable to pinpoint the exact drivers of the observed changes. Additionally, as stated in the methods, subjects reported that they were healthy but were not screened for any diagnosed conditions. We feel this best represents a real-world consumer likely to seek sleep support.

Considering the complex combinations of products examined in this study, further investigations are warranted to identify the specific active ingredient(s) responsible for the observed significant effects. To achieve this, a rigorous study employing a factorial design with multiple arms would be beneficial. This design would systematically vary the ingredients in different combinations, allowing for the examination of both their individual effects and potential interactions on behavioral outcomes. Such an approach would enhance our understanding of the precise drivers behind the observed effects.

## 5. Conclusions

In this randomized, double-blind, placebo-controlled trial to evaluate the effects of two formulations of sleep softgels relative to placebo, we observed that a botanical blend containing lower amounts of THC and higher amounts of GABA, hops oil, and valerian oil significantly improved sleep quality, anxiety, stress, and overall well-being in healthy individuals with a desire for better sleep. We observed no significant difference in effect on any health outcome (sleep quality, anxiety, stress, pain, or overall well-being) between a botanical blend containing higher amounts of THC and lower amounts of GABA, hops oil, and valerian oil and the placebo control. The active products demonstrated a favorable safety profile; all side effects were mild or moderate, and there were no significant differences in the frequency of reported side effects between the active arms and placebo.

## Figures and Tables

**Table 1:** Validated measures for key outcomes used in Radicle Rest study.

Measure	Description	Scoring Interpretation	How Was This Collected?
PROMIS Sleep Disturbance 8a	Eight-item measure assessing sleep disturbance (sleep quality) in the past 7 days.	Scoring from 8 to 40, with higher scores translating to greater sleep disturbance.	All participants received this measure within their weekly health surveys.
PROMIS Anxiety 4a	Four-item measure assessing frequency of anxiety symptoms in the past 7 days.	Scoring from 4 to 20, with higher scores translating to greater anxiety.	Participants who endorsed anxiety symptoms received this measure in their weekly health surveys.
PROMIS Stress 4a	Four-item measure assessing frequency of stress symptoms in the past 7 days.	Scoring from 4 to 20, with higher scores translating to greater stress.	Participants who endorsed stress symptoms received this measure in their weekly health surveys.
PEG (Pain, Enjoyment, General Activity) scale	Three-item measure assessing pain intensity and interference in the past 7 days.	Scoring from 0 to 10, with higher scores translating to greater pain.	Participants who endorsed pain symptoms received this measure in their weekly health surveys.
World Health Organization (WHO)-5 Well-being index	Five-item measure assessing feelings of well-being in the past 7 days.	Scoring from 0 to 25, with higher scores translating to greater well-being.	All participants received this measure within their weekly health surveys.

**Table 2:** Participant sample summary at baseline.

Variable	Placebo	Arm 1	Arm 2	
	Mean (SD)/N (%)	Mean (SD)/N (%)	Mean (SD)/N (%)	t/χ^2^ *p*-Value
N	207	206	207	
Age	44.51 (11.08)	45.08 (11.00)	45.35 (10.86)	0.73
Race				0.307
White	177 (85.5)	162 (78.6)	167 (80.7)	
Non-white	29 (14.0)	44 (21.4)	39 (18.8)	
Native Hawaiian or Pacific Islander	1 (0.5)	0 (0.0)	1 (0.5)	
Education: no college degree	96 (46.4)	116 (56.3)	97 (46.9)	0.075
Sex at birth: male	71 (34.3)	71 (34.5)	71 (34.3)	0.999
Hispanic, LatinX, or Spanish origin: yes	19 (9.2)	20 (9.7)	17 (8.2)	0.865
BMI	29.27 (7.95)	30.59 (8.10)	30.17 (8.00)	0.233
PROMIS sleep disturbance 8A	28.82 (6.22)	29.85 (6.59)	29.65 (6.39)	0.22
PROMIS anxiety 4A	10.46 (3.47)	11.02 (3.60)	11.06 (3.35)	0.145
PROMIS stress 4A	12.81 (3.42)	13.36 (3.76)	13.39 (3.55)	0.176
Pain, enjoyment, general activity scale	5.60 (2.14)	5.87 (2.30)	5.68 (2.10)	0.618

**Table 3:** Significant factors associated with sleep disturbance: results from a linear mixed-effects regression model. Summary of significant variables and their associations with sleep disturbance based on a linear mixed-effects regression model. The model was used to assess the differences in the change in the variables of interest between each active product arm versus placebo. The table presents the beta coefficients (β), standard errors (Std.Error), degrees of freedom (DF), t-values, and *p*-values for each variable. Higher values indicate a stronger positive association with sleep disturbance, while lower values indicate a stronger negative association.

	Value	Std.Error	DF	t-Value	*p*-Value
(Intercept)	24.725	1.367	1238	18.089	<0.001
Education	1.846	0.477	610	3.872	<0.001
Sex at Birth	−0.075	0.492	610	−0.153	0.879
Age	−0.018	0.022	610	−0.822	0.411
Race: non-white	−0.933	0.615	610	−1.517	0.130
Race: Native Hawaiian or Pacific Islander	3.028	3.833	610	0.790	0.430
BMI	0.070	0.029	610	2.408	0.016
Hispanic, LatinX, or Spanish origin	0.152	0.830	610	0.184	0.854
Study week	−1.339	0.150	1238	−8.919	<0.001
TreatSleep A (Arm 1)	0.424	0.592	610	0.715	0.475
TreatSleep B (Arm 2)	0.458	0.586	610	0.781	0.435
Study week: TreatSleep A (Arm 1)	−0.639	0.212	1238	−3.010	0.003
Study week: TreatSleep B (Arm 2)	−0.236	0.208	1238	−1.133	0.257

**Table 4:** Significant factors associated with anxiety: results from a linear mixed-effects regression model. Summary of significant variables and their associations with anxiety based on a linear mixed-effects regression model. The model was used to assess the differences in the change in the variables of interest between each active product arm versus placebo. The table presents the beta coefficients (β), standard errors (Std.Error), degrees of freedom (DF), t-values, and *p*-values for each variable. Higher values indicate a stronger positive association with anxiety, while lower values indicate a stronger negative association.

	Value	Std.Error	DF	t-Value	*p*-Value
(Intercept)	11.007	0.764	773	14.415	<0.001
Education: no college degree [Ref: College Degree]	0.744	0.266	610	2.795	0.005
Sex at birth: male [Ref: Female]	−0.398	0.276	610	−1.439	0.151
Age	−0.035	0.012	610	−2.855	0.005
Race: Non-white [Ref: white]	−0.027	0.345	610	−0.078	0.938
Race: Native Hawaiian or Pacific Islander [Ref: white]	0.667	2.258	610	0.295	0.768
BMI	0.021	0.016	610	1.264	0.207
Hispanic, LatinX, or Spanish origin: yes [Ref: No]	0.196	0.452	610	0.434	0.665
Study week	−0.445	0.087	773	−5.126	<0.001
Sleep A (Arm 1)	0.388	0.329	610	1.179	0.239
Sleep B (Arm 2)	0.577	0.326	610	1.769	0.078
Study week: Sleep A (Arm 1)	−0.259	0.127	773	−2.044	0.041
Study week: Sleep B (Arm 2)	−0.066	0.123	773	−0.537	0.592

**Table 5:** Significant factors associated with stress: results from a linear mixed-effects regression model. Summary of significant variables and their associations with stress based on a linear mixed-effects regression model. The model was used to assess the differences in the change in the variables of interest between each active product arm versus placebo. The table presents the beta coefficients (β), standard errors (Std.Error), degrees of freedom (DF), t-values, and *p*-values for each variable. Higher values indicate a stronger positive association with stress, while lower values indicate a stronger negative association.

	Value	Std.Error	DF	t-Value	*p*-Value
(Intercept)	14.746	0.782	835	18.856	<0.001
Education: no college degree [Ref: College Degree]	0.435	0.272	610	1.597	0.111
Sex at birth: male [Ref: Female]	−0.666	0.283	610	−2.353	0.019
Age	−0.065	0.012	610	−5.239	<0.001
Race: non-white [Ref: white]	−0.287	0.352	610	−0.814	0.416
Race: Native Hawaiian or Pacific Islander [Ref: white]	−0.108	2.331	610	−0.046	0.963
BMI	0.033	0.017	610	1.971	0.049
Hispanic, LatinX, or Spanish origin: Yes [Ref: No]	−0.107	0.471	610	−0.228	0.820
Study week	−0.521	0.088	835	−5.913	<0.001
Sleep A (Arm 1)	0.202	0.334	610	0.605	0.545
Sleep B (Arm 2)	0.501	0.332	610	1.507	0.132
Study week: Sleep A (Arm 1)	−0.360	0.126	835	−2.864	0.004
Study week: Sleep B (Arm 2)	−0.047	0.125	835	−0.372	0.710

**Table 6:** Significant factors associated with pain: results from a linear mixed-effects regression model. Summary of significant variables and their associations with pain based on a linear mixed-effects regression model. The model was used to assess the differences in the change in the variables of interest between each active product arm versus placebo. The table presents the beta coefficients (β), standard errors (Std.Error), degrees of freedom (DF), t-values, and *p*-values for each variable. Higher values indicate a stronger positive association with pain, while lower values indicate a stronger negative association.

	Value	Std.Error	DF	t-Value	*p*-Value
(Intercept)	3.078	0.647	689	4.760	<0.001
Education: no college degree [Ref: College Degree]	0.706	0.230	346	3.076	0.002
Sex at birth: male [Ref: Female]	−0.016	0.241	346	−0.067	0.946
Age	0.024	0.010	346	2.355	0.019
Race: Non-white [Ref: white]	0.014	0.297	346	0.046	0.964
BMI	0.030	0.013	346	2.304	0.022
Hispanic, LatinX, or Spanish origin: yes [Ref: No]	0.216	0.458	346	0.471	0.638
Study week	−0.307	0.063	689	−4.875	<0.001
Sleep A (Arm 1)	−0.073	0.273	346	−0.267	0.790
Sleep B (Arm 2)	−0.155	0.274	346	−0.566	0.572
Study week: Sleep A (Arm 1)	−0.024	0.087	689	−0.270	0.788
Study week: Sleep B (Arm 2)	0.032	0.088	689	0.368	0.713

**Table 7:** Significant factors associated with overall well-being: results from a linear mixed effects regression model. Summary of significant variables and their associations with overall well-being based on a linear mixed-effects regression model. The model was used to assess the differences in the change in the variables of interest between each active product arm versus placebo. The table presents the beta coefficients (β), standard errors (Std.Error), degrees of freedom (DF), t-values, and *p*-values for each variable. Higher values indicate a stronger positive association with overall well-being, while lower values indicate a stronger negative association.

	Value	Std.Error	DF	t-Value	*p*-Value
(Intercept)	11.251	1.020	1238	11.031	<0.001
Education: no college degree [Ref: College Degree]	−1.559	0.356	610	−4.383	<0.001
Sex at birth: male [Ref: Female]	0.315	0.368	610	0.856	0.392
Age	0.015	0.016	610	0.898	0.369
Race: non-white [Ref: white]	0.781	0.458	610	1.706	0.089
Race: Native Hawaiian or Pacific Islander [Ref: white]	1.657	2.918	610	0.568	0.570
BMI	−0.060	0.022	610	−2.752	<0.001
Hispanic, LatinX, or Spanish origin: yes [Ref: No]	0.266	0.616	610	0.432	0.666
Study week	0.672	0.106	1238	6.308	<0.001
Sleep A (Arm 1)	0.422	0.431	610	0.980	0.327
Sleep B (Arm 2)	0.084	0.427	610	0.196	0.844
Study week: Sleep A (Arm 1)	0.318	0.151	1238	2.116	<0.001
Study week: Sleep B (Arm 2)	−0.005	0.148	1238	−0.037	0.971

## Data Availability

The data presented in this study are available on request from the corresponding author. The data are not publicly available.
